# A genetic variant of the atrial natriuretic peptide gene is associated with left ventricular hypertrophy in a non-diabetic population – the Malmö preventive project study

**DOI:** 10.1186/1471-2350-14-64

**Published:** 2013-06-24

**Authors:** Amra Jujić, Margret Leosdottir, Gerd Östling, Petri Gudmundsson, Peter M Nilsson, Olle Melander, Martin Magnusson

**Affiliations:** 1 Department of Biomedical Laboratory Science, Malmö University, Malmö, Sweden; 2 Department of Cardiology, Skåne University Hospital Malmö, and Lund University, Malmö, Sweden; 3 Department of Clinical Sciences, Lund University, Skåne University Hospital Malmö, Malmö, Sweden; 4 Center of Emergency Medicine, Skåne University Hospital Malmö, Malmö, Sweden

## Abstract

**Background:**

Epidemiological studies have shown considerable heritability of blood pressure, thus suggesting a role for genetic factors. Previous studies have shown an association of a single nucleotide polymorphism *rs5068* in the NPPA locus gene with higher levels of circulating atrial natriuretic peptide as well as with lower intra individual blood pressure, but up to date, no association between *rs5068* and cardiac organ damage, i.e. left ventricular hypertrophy, has been accounted for in humans. We sought to explore if *rs5068* is associated with left ventricular hypertrophy as measured by echocardiographic examination in a non-diabetic population.

**Methods:**

968 non-diabetic individuals from the Malmö Preventive Project (mean age 67 years; 31% women) were genotyped and examined with echocardiography. Logistic regression was used to adjust for covariates.

**Results:**

The minor allele of *rs5068* was associated with decreased prevalence of left ventricular hypertrophy (p = 0.021) after adjustment for sex and age. In the multivariate logistic analysis including; age, sex, systolic blood pressure, antihypertensive and/or cardioprotective treatment, body mass index and fasting plasma glucose, the association of *rs5068* with left ventricular hypertrophy was, as expected, attenuated (p = 0.061).

**Conclusion:**

In a non-diabetic population, the minor allele of *rs5068* was associated with lower left ventricular mass. These findings suggest that *rs5068*, or genetic variants in linkage disequilibrium, might affect susceptibility to left ventricular hypertrophy and support the possible protective role of natriuretic peptides.

## Background

Atrial natriuretic peptide (ANP) and brain natriuretic peptide (BNP) are secreted from cardiomyocytes in response to cardiac wall stress and have important roles in the regulation of extracellular fluid volumes and blood pressure (BP) [1-3]. Animal studies have shown that over expression of natriuretic peptide genes is associated with low BP, whereas under expression is associated with high BP and left ventricular hypertrophy (LVH) [4,5], and studies on mice with inactivated type A natriuretic peptide receptors (NPRA) have even shown hypertrophic growth of the heart independently of BP levels [6]. The suppressive effect of ANP on hypertrophy has also been demonstrated in cardiomyocytes in culture [7] and a recent study showed salt-sensitive hypertension in natriuretic peptide precursor A (NPPA) knockout mice, demonstrating that genetically reduced production of ANP can lead to salt-sensitive hypertension [8]. Evidence of substantial heritability of plasma natriuretic peptide (NP) levels has also been accounted for [9] and low circulating NP levels have been associated with obesity [10] and new onset diabetes [11], which may contribute to their predisposition to hypertension.

The NPPA and natriuretic peptide precursor B (NPPB) genes lie in tandem 9.7 kb apart on chromosome 1. Associations between common variations (*rs5068*, *rs198358* and *rs632793*) in NPPA-NPPB locus genes and higher levels of circulating ANP were found in a recent study as well as association with lower interindividual BP, with the strongest effect for *rs5068*[12], findings that have been replicated in larger cohorts [13]. The minor allele of *rs5068* has also been associated with favorable cardiometabolic profile [14], and there is evidence for an important protective role of the natriuretic peptides in individuals with coronary artery disease [15], but surprisingly no association with echocardiographic evidence of cardiac organ damage, such as LVH, for this particular SNP. However, since LVH is well known to be overrepresented in patients with diabetes mellitus [11,16-19] the inclusion of subjects with diabetes, 17% and 15% respectively) might explain the lack of association of *rs5068* and LVH [14,15].

Here we tested the hypothesis that *rs5068* suppressively regulates the development of LVH in a non-diabetic population.

## Methods

### Study sample

Between 1974–1992, birth cohorts (men born in 1921, 1926–1942, 1944, 1946 and 1948–9; women born in 1926, 1928, 1930–6, 1938, 1941–2 and 1949) of inhabitants in Malmö, Sweden, were invited to participate in a large cohort study The Malmö Preventive Project (MPP) with a total of 33,346 individuals attending (67% men, attendance rate 71%, mean age 46 ± 7 years). Re-examination of 18,240 surviving MPP participants, the MPP-Re-examination Study (MPP-RES) was conducted during 2002–2006 (63% men, 72% attendance rate, mean age 69 ± 6 years). All participants answered a self-administered questionnaire on lifestyle and medical history. Height and weight were measured in light in-door clothing, waist circumference was measured and body mass index (BMI) was calculated (kg/m^2^). Patient BP and pulse rate were measured twice in the supine position after 10 minutes of rest and blood samples were drawn after an overnight fast. In a sample of 1,792 participants, echocardiography (UCG) and electrocardiography (ECG) recordings were carried out. These subjects were randomly selected from groups defined by glucometabolic status: normal fasting plasma glucose (FPG) (≤6.0 mmol/l); impaired FPG (IFG); new-onset type 2 diabetes mellitus; and prevalent diabetes mellitus; with oversampling in groups of subjects with glucometabolic disturbances to ensure sufficient numbers of subjects studied from each group. A full description of this study population has been presented elsewhere [20]. Subjects with prevalent diabetes mellitus type 1 or 2 (n = 677) were excluded from our analysis due to previously shown overrepresentation of diastolic dysfunction and LVH within the diabetic phenotype [11,16,17], resulting in a total of 1,115 subjects. Of these 1,115 subjects, 147 had missing values of one or more covariates used in the multiple regression analysis, resulting in 968 eligible subjects.

All participants signed a written informed consent form before entering the MPP-RES. The study was approved by The Ethics committee at Lund University, Sweden.

### Echocardiography

Conventional echocardiograms were obtained with an Acuson Sequoia, Mountain View, CA, USA (3V2c transducer) or Sonos 5500 Philips, Andover, MA, USA (S3 transducer). All echocardiograms were carried out by experienced sonographers. Internal left ventricular dimensions were evaluated from parasternal long axis view according to the recommendations of the European Association of Echocardiography. Measurements of wall thickness were obtained in two-dimensional end-diastolic parasternal long axis view, where left ventricular mass (LVM) was calculated during a single heart cycle if recordings were homogeneous, otherwise a mean of three to five measurement of different heart cycles was used. LVM was calculated according to clinical standards and indexed for height^2.7^ to minimize the effect of obesity on LVM calculations [21].

Two sonographers analysed images independently from a random sub-sample of subject to test inter- and intra-observer variability which was 13.0% and 10.5% for intraventricular end-diastolic septum diameter, and 4.1% and 3.3% for left ventricular end-diastolic diameter. Both sonographers were experienced and blinded to clinical data. All analyses were performed offline using Xcelera software (Philips).

### Genotyping

Variants in NPPA-NPPB locus genes were analysed by direct genotyping as described previously using TaqMan (Applied Biosystems) with primers and conditions as recommended by the manufacturer [12]. Twenty five percent of the samples were run in duplicates without discrepancies.

### Laboratory assays

All FPG analyses were performed by Department of Clinical Chemistry, Malmö University Hospital which is attached to a national standardization and quality control system (Beckman Coulter LX20, Beckman Coulter Inc, Brea, USA).

### Other definitions

If LVM was >51 g/m^2.7^, LVH was considered prevalent [21,22]. Antihypertensive and/or cardioprotective treatment was defined as use of one or more of the following; β-receptor blockers, calcium channel blockers, lipid lowering drugs, angiotensin converting enzyme inhibitors, long-acting nitrates, angiotensin receptor blockers, digitalis and diuretics. Prevalent hypertension was defined as the presence of either one or more of the following; systolic BP (SBP) > 140 mmHg and/or diastolic BP >90 mmHg and/or antihypertensive and/or treatment by the time of the third visit. Diabetes was defined as previously known diabetes mellitus type 1 or 2, whereas new-onset diabetes mellitus type 2 was defined by either two separate measurements of FPG ≥7.0 mmol/l, or one single measurement of ≥11.1 mmol/l.

### Statistical analysis

Continuous variables were expressed as mean ± standard deviation (SD). FPG was not normally distributed and was log transformed prior to analysis. LVM was modeled as the dependent variable in linear regression and the presence of LVH via logistic regression with *rs5068* genotype as independent variable using additive models with the minor allele (G) coded. All modeling was performed in models adjusted for age and sex (model 1) and for age, sex, BMI, SBP, antihypertensive and/or cardioprotective treatment, and FPG (model 2). A two-tailed significance level of p < 0.05 was considered statistically significant. All calculations were done in SPSS 19.0 (SPSS Inc, Illinois, USA).

## Results

Baseline characteristics for the entire non diabetic population and subgroup characteristics (subjects without, and subjects with LVH) are presented in Table 1. The mean age of participants was 67.1 years and 30.5% of subjects were female. Subjects with LVH were older (p < 0.001), with higher BMI (p < 0.001), SBP (p = 0.001) and higher prevalence of hypertension (p < 0.001) compared to subjects without LVH. Genotype frequencies of *rs5068* are presented in Table 2. LVM index for the total population, subjects without LVH and subjects with LVH is presented in Table 3.

**Table 1:** Baseline characteristics of the study population

	**Total sample**	**Subjects without LVH**	**Subjects with LVH**
**N**	968	820	148
**Sex (% women)**	30.5	30.6	29.7
**Age (years)**	67.1 (±5.7)	66.7 (±5.7)	69.4 (±5.2)
**Systolic BP (mmHg)**	146.0 (±19.3)	145.1 (±19.0)	150.8 (±20.2)
**Diastolic BP (mmHg**)	84.5 (±10.2)	84.2 (±10.0)	86.6 (±11.3)
**Antihypertensive and**/**or cardioprotective treatment** (%)	41.3	37.8	60.8
**Hypertension** (%)	74.5	71.5	91.1
**BMI**	27.3 (±3.8)	26.9 (±3.4)	29.8 (±4.8)
FPG (mmol/l)*	5.8 [0.9]	5.8 [1.0]	6.1 [1.0]

Data are expressed as mean (±SD) or median [interquartile range (IQR)]*. *BP* blood pressure, *BMI* body mass index, *FPG* fasting plasma glucose.

**Table 2:** Genotype frequencies of rs5068

	**Total sample**		**Subjects without LVH**		**Subjects with LVH**	
	**Frequency (n)**	**Percent (%)**	**Frequency (n)**	**Percent (%)**	**Frequency (n)**	**Percent (%)**
AA	847	87.5	710	86.6	137	92.6
AG	117	12.1	106	12.9	11	7.4
GG	4	0.4	4	0.5	0	0
Total	968	100	820	100	148	100

Genotype frequencies of rs5068 in total sample, subjects without diabetes and subjects with diabetes. Major allele represented by A, minor allele represented by G.

**Table 3:** **Left ventricular mass index (g/m**^**2.7**^**)**

**LVM index (g/m**^**2.7**^**)**	**n**	**Min**	**Max**	**Mean**	**SE**
**Total population**	968	18.96	99.31	40.44	11.28
**Subjects without LVH**	820	18.96	50.97	36.86	7.13
**Subjects with LVH**	148	51.00	99.31	60.21	9.36

Values are beta-coefficients (standard errors).

LVM index for the total population, for patients with and patients without LVH.

In logistic regression analysis, the *rs5068* minor allele was significantly associated with lower prevalence of LVH (p = 0.021) (Table 4). However, after adjustment according to model 2 (age, sex, SBP, antihypertensive and/or cardioprotective treatment, BMI and logFPG) this correlation was attenuated (p = 0.061) (Table 5). Results for Hosmer-Lemeshow goodness of fit test as well as sensitivity, specificity and percent of overall correct for model 2 are presented in Table 6.

**Table 4:** Association of *rs5068 *with LVH in a non diabetic population adjusted for age and gender*

	**B**	**SE**	**Wald**	**P-value**	**OR (95% CI)**
**Gender**	−0.331	0.203	0.656	0.103	0.72 (0.48-1.07)
**Age**	0.095	0.017	31.205	<0.001	1.10 (1.06-1.14)
**rs5068**	−0.757	0.328	5.311	0.021	0.47 (0.25-0.89)

*Model 1: Age and sex adjusted.

Values are odds ratios (95% confidence intervals) for prevalent LVH. SE values are standard errors. B values are unstandardized regression coefficients.

**Table 5:** Association of *rs5068 *with LVH in a non diabetic population in a fully adjusted model*

	**B**	**SE**	**Wald**	**p-value**	**OR (95% CI)**
**Gender**	−0.460	0.225	4.165	0.041	0.63 (0.41-0.98)
**Age**	0.101	0.019	29.714	<0.001	1.12 (1.07-1.15)
**rs5068**	−0.630	0.337	3.504	0.061	0.53 (0.28-1.03)
**BP**	0.010	0.005	4.590	0.032	1.01 (1.00-1.02)
**logFPG**	0.177	0.867	0.042	0.838	1.19 (0.22-6.53)
**AHT**	0.525	0.202	6.789	0.009	1.69 (1.14-2.51)
**BMI**	0.191	0.027	48.988	<0.001	1.21 (1.15-1.28)

*Model 2: Age, sex, BP, AHT, logFPG and BMI adjusted.

Values are odds ratios (95% confidence intervals) for prevalent LVH. SE values are standard errors. B values are unstandardized regression coefficients.

**Table 6:** Hosmer-Lemeshow goodness of fit test, sensitivity, specificity and percent of overall correct for association of *rs5068 *and LVH in a fully adjusted model*

**Number of observations**	968
**Number of groups**	10
**Hosmer-Lemeshow chi2(8)**	10.42
**Prob > chi2**	0.2369
**Sensitivity**	15.54%
**Specificity**	98.90%
**Correctly classified**	86.16%

*Model 2: Age, sex, BP, AHT, logFPG and BMI adjusted.

In linear regression analysis, the minor allele of *rs5068* was significantly associated with decreased LVM indexed for height^2.7^ after adjustment according to model 1 (p = 0.041) (Table 7), however after full multivariate adjustment according to model 2 this association was no longer significant (p = 0.165) (Table 8). Seven point six percent of the variation in the response variable can be explained by the explanatory variables. Hardy Weinberg equilibrium significance analysis did not show any significant deviation (p-value = 0.117).

**Table 7:** **Association of *rs5068 *with LVM Indexed for Height**^**2.7 **^in a non Diabetic Population adjusted for age and sex*

	**B**	**SE**	**Beta**	**p-value**	**Adjusted R Square**	**F**
**Age**	0.567	0.064	0.287	<0.001		
**rs5068**	−2.060	1.006	−0.063	0.041		
**Gender**	−3.126	0.794	−0.127	<0.001		
**Full equation**				<0.001	0.076	27.645

*Model 1. Adjusted for age and sex.

SE values are standard errors. Beta values are standardized regression coefficients. B are unstandarized coefficients.

**Table 8:** **Association of *rs5068 *with LVM Indexed for Height**^**2.7 **^in a non diabetic population in a fully adjusted model*

	**B**	**SE**	**Beta**	**p-value**	**Adjusted R Square**	**F**
**BP**	0.053	0.017	0.090	0.002		
**FPG**	−1.323	2.937	−0.014	0.653		
**Age**	0.537	0.060	0.272	<0.001		
**rs5068**	−1.282	0.922	−0.039	0.165		
**BMI**	1.033	0.089	0.347	<0.001		
**AHT**	2.140	0.678	0.093	0.002		
**Gender**	−3.274	0.747	−0.134	<0.001		
**Full equation**				<0.001	0.228	41.754

*Model 2: Age, sex, BP, AHT, logFPG and BMI adjusted. SE values are standard errors. Beta values are standardized regression coefficients.

## Discussion

The key finding of our study is that the minor allele of *rs5068* is associated with lower LVM as well as lower prevalence of LVH in a non-diabetic population after adjustments for age and sex.

These associations were attenuated in the multivariate analysis in which SBP and antihypertensive and/or cardioprotective treatment were included. One possible explanation for this could be that the observed association between the minor allele of *rs5068* and lower likelihood of LVH is explained by lower BP mediated by the same polymorphism [12]. On the other hand, the attenuation of the association between *rs5068* and LVH after full adjustment was rather modest, suggesting that the ANP elevating effects of the *rs5068* G-allele also may contribute with BP independent effects on LVH. We acknowledge that the effect size is small, as commonly the case is in polygenic situations. However, the effect size for most variants is small and a single SNP can only explain a small proportion of the expected heritability.

In contrast to the results of Cannone and co-workers [14], but also Ellis and co-workers [15], we were able to show a significant association between the minor allele of *rs5068* and reduced prevalence of LVH, which can be explained by our exclusion of subjects with diabetes. LVH is heavily overrepresented in patients with diabetes [19,23] and the existence of a specific diabetic cardiomyopathy has been an ongoing debate over the past decades [24]. It is likely to presume that the “harmful” diabetic phenotype overrides the cardioprotective and/or the blood pressure lowering effects of the minor allele of *rs5068*. Also, in comparison of clinical studies of LVH, adequate correction is of critical importance, since both body habitus and size are associated with LV mass and dimensions. Normalization of LV mass is still a subject for debate, since different criteria for body size adjustment result in different prevalence of patients with LVH. The body surface area correction has been shown to underestimate LVM in the upper range of body surface area distribution, and a height-based adjustment has been proposed to correct for obesity. The most accurate estimation of LVH appears to be derived from indexing for height^2.7^, where 51 g/m^2.7^ appears to be a reliable cut off value to define LVH, as used in our study. [21] As both Cannone and Ellis corrected for body surface area, our results may not be fully comparable.

Echocardiography is the most commonly used imaging modality in assessing LVM, left ventricular size and performance. However, it is subjective and operator dependent and contends with inter- and intraobserver variability, particularly when using harmonic imaging in assessing LVM. Even though harmonic imaging tends to overestimate wall thickness, it has also improved delineation of the left ventricle, especially in subjects with bad image quality [25]. All methods for quantitation of LVM using two-dimensional imaging have been validated previously [26,27]. We sought to minimize the effects of subjectivity on our data by testing if inter- and intraobserver variability between two independent readers analysing images was within acceptable levels.

As our sample size was relatively small, the results warrant replication in a larger cohort.

As this is a cross-sectional study, it shares the inherent limitations about causality and control as all cross-sectional studies. Still, the genetic effect is not expected to be hampered by such confounding, as the genome is constant throughout life.

Our data was collected at a single regional centre which limits the applicability to other populations and allows the influence of community characteristics. On the other hand, inter-observer bias regarding the UCG measurement was less than would be expected in a multi-centre study. Also, although the subjects with congestive heart failure were overrepresented in the original population, excluding subjects with diabetes resulted in low number of subjects with congestive heart failure (n = 4). In order to prove that inclusion of those subjects does not have any impact on our data, we performed a sensitivity analysis by excluding patients with prevalent heart failure and received very similar data (not shown). Hence, inclusion of patients with congestive heart failure is not considered to have any impact on our results.

Finally, our study results may not be generalizable to all ancestries since the study was undertaken in mainly individuals of Swedish descent.

## Conclusions

In a non-diabetic population, the minor allele of *rs5068* was associated with lower LVM and lower prevalence of LVH. Whether the association is mediated by the BP lowering effect of *rs5068* or is independent of BP is uncertain. However, our findings suggest that *rs5068* or genetic variants in linkage disequilibrium might affect susceptibility for LVH and support the possible protective role of NP.

## Competing interests

The authors declare that they have no competing interests.

## Authors’ contributions

Drs MM, OM, ML and AJ, have all participated in the conception and design of the study and all authors (Drs MM, OM, ML, PN, GÖ, PG and AJ) have contributed in the analysis and interpretation of data and also in the drafting and revising of the manuscript. Before submission each author have read and thereafter given his final approval of the manuscript. The manuscript has not been published and is not being considered for publication in whole or part in any language.

## Pre-publication history

The pre-publication history for this paper can be accessed here:

http://www.biomedcentral.com/1471-2350/14/64/prepub
